# Die atrioösophageale Fistel nach Pulmonalvenenisolation – eine interdisziplinäre Herausforderung

**DOI:** 10.1007/s00101-023-01316-x

**Published:** 2023-07-25

**Authors:** Caroline Neumann, Christian von Loeffelholz, Gloria Färber, Kathleen Lange, Michael Bauer

**Affiliations:** 1 Klinik für Anästhesiologie und Intensivmedizin, Universitätsklinikum Jena, Am Klinikum 1, 07747 Jena, Deutschland; 2 Klinik für Herz- und Thoraxchirurgie, Universitätsklinikum Jena, Am Klinikum 1, 07747 Jena, Deutschland; 3 Klinik für Innere Medizin IV – Gastroenterologie, Hepatologie, Infektiologie, Universitätsklinikum Jena, Am Klinikum 1, 07747 Jena, Deutschland

## Anamnese

Ein 47-jähriger männlicher Patient (BMI [„body mass index“] 37,2 kg/m^2^) mit vorbestehendem arteriellen Hypertonus, persistierendem Vorhofflimmern, chronischem Nikotinabusus und Schlafapnoesyndrom wurde mit einer Hypästhesie des linken Arms und hohem Fieber in der Notaufnahme eines externen Krankenhauses vorstellig. Seine Dauermedikation bestand aus Bisoprolol, Amlodipin, Lisinopril, Digitoxin und Edoxaban. Vor einem Monat wurde eine Lungenvenenisolation mit Substratmodifikation als „Hitzeablation“ unter Verwendung eines 3D-Mapping des Herzens mit Fluoroskopieintegration und kontinuierlicher Messung des Anpressdruckes (CARTO-UNIVU, Biosense Webster, Irvine, CA, USA) als Ersteingriff durchgeführt.

## Befund

Im Rahmen der klinischen Untersuchung war der Patient in einem signifikant reduzierten Allgemeinzustand, jedoch hämodynamisch und respiratorisch stabil. Das EKG zeigte eine Tachyarrhythmie von 130–140 bpm („beats per minute“).

## Diagnostik

Laborchemisch zeigten sich neben deutlich erhöhten Entzündungsparametern mit gesteigertem CRP (200 mg/l) und PCT (17,5 μg/l) eine bis dato unbekannte Nierenfunktionsstörung (GFR 36 ml/min nach CKD-EPI).

Bei Vorhofflimmern wurde zunächst eine kardioembolische Genese des neurologischen Defizits angenommen, die sich mittels zerebraler computertomographischer Angiographie (CTA) aber nicht bestätigen ließ.

Im Rahmen der Abklärung der erhöhten Entzündungsparameter wurden periphere Blutkulturen entnommen, die den Nachweis von *Streptococcus salivarius* erbrachten.

Während der transösophagealen Echokardiographie (TEE) wurden temporäre Lufteinschlüsse im Bereich des linken Vorhofs festgestellt, sodass nun der dringende Verdacht auf eine atrioösophageale Fistel bestand.

## Therapie und Verlauf

**Figure 1:**
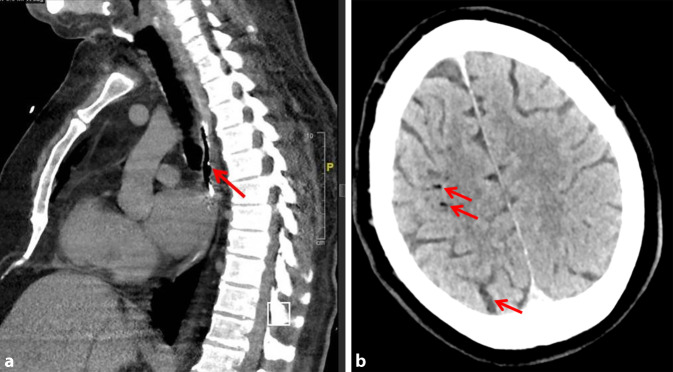

Eine empirische Therapie mit Piperacillin/Tazobactam und Caspofungin wurde begonnen und der Patient in das Universitätsklinikum Jena verlegt. Ergänzend erfolgte eine thorakale Kontrastmittel-CT, die mit dorsal des linken Vorhofs befindlichen Lufteinschlüssen (Abb. 1a) die Diagnose bestätigte.

Im weiteren Verlauf kam es zu einer hämodynamischen Verschlechterung, und neben der Gabe von Kristalloiden wurde eine Vasopressortherapie mit Noradrenalin (maximal 0,16 μg/kgKG und min, Lactat maximal 2,7 mmol/l) initiiert.

Eine stark wechselnde neurologische Symptomatik (schlaffe armbetonte Hemiparese links, Herdblick nach rechts, Feinmotorikstörung der kontralateralen Hand und eine latente rechtsseitige Hemiparese sowie Dysarthrie) führte zu einer erneuten CCT, die mehrere kortikale Lufteinschlüsse zeigte (Abb. 1b).

**Figure 2:**
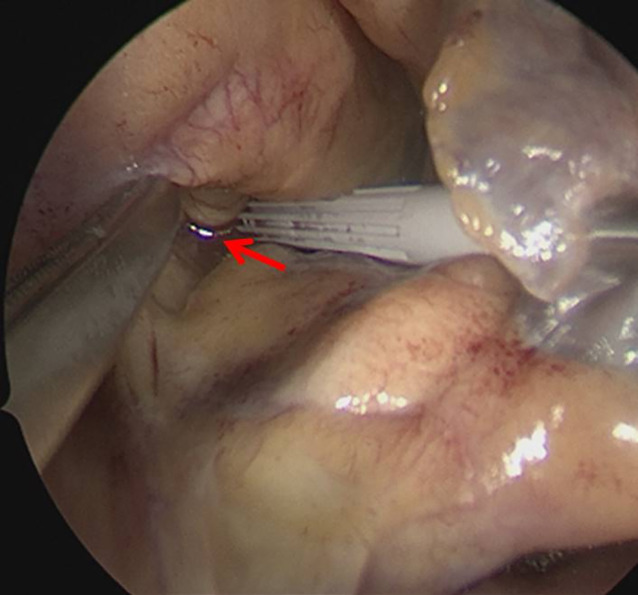

Die weitere Therapie des Patienten erfolgte im engmaschigen Dialog und Konsens der verschiedenen Fachabteilungen – der Herz‑/Thoraxchirurgie, der Intensivmedizin und den endoskopisch tätigen Internisten. Da das Aspirationsrisiko im Rahmen der Narkoseinduktion als sehr hoch eingeschätzt wurde, erfolgte eine „rapid sequence induction“ mit dem Ziel einer möglichst schnellen Sicherung des Atemweges über die endotracheale Intubation. Nach Präoxygenierung mit 15 l Sauerstoff über eine dichtsitzende Maske sowie rascher Applikation der Opioide, Sedativa und schnell wirksamen Muskelrelaxanzien erfolgte die problemlose Intubation ohne Zwischenbeatmung. Bei einem sichtbaren Defekt zwischen den oberen Pulmonalvenen und der Vorhofhinterwand (Abb. 2) entschied man sich für ein primär operatives Vorgehen durch Patch-Plastik mit Vorhofohrverschluss. Der Eingriff wurde in Allgemeinanästhesie unter Einsatz der Herz-Lungen-Maschine durchgeführt. Der Patient erhielt zwei rechtsseitige Pleura- und eine Jackson-Pratt-Drainage, die intraoperativ hinter dem Patch positioniert und postoperativ für insgesamt 3 Tage mit Polyhexanid und Polyethylenglycol gespült wurden.

**Figure 3:**
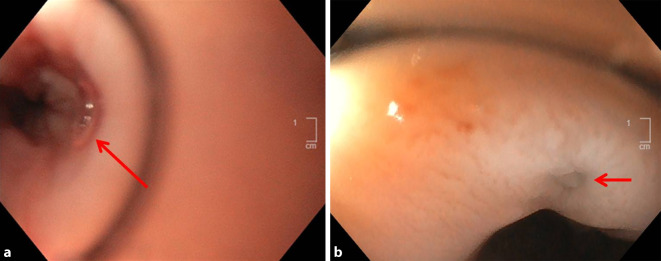

Sekundär erfolgte unmittelbar postoperativ die endoskopische Anlage einer Foliensaugdrainage (Trelumina mit Folie) im Bereich der bei 33 cm ab Zahnreihe nachweisbaren ösophagealen Fistelöffnung (Abb. 3a) mit dem Ziel, neben der operativen Reparatur des Defektes im linken Vorhof, auch den ösophagealen Defekt über eine Sekretdrainage zu adressieren (kombinierte Reparatur).

Aufgrund oraler Nahrungskarenz erfolgte die enterale Ernährung über die eingebrachte Treluminalsonde.

In einer endoskopischen Verlaufskontrolle nach 6 Tagen zeigte sich die Fistel komplett verschlossen. Lediglich eine kleine residuelle kreisrunde Narbe war zu sehen (Abb. 3b)*.* Die Treluminalsonde konnte entfernt und eine selbstständige orale Nahrungsaufnahme begonnen werden.

Vierzehn Tage nach erfolgreicher Operation wurde der Patient ohne Residuen in die neurologische Frührehabilitation verlegt.

## Diskussion

Die Manifestation der atrioösophagealen Fistel nach Pulmonalvenenisolation zeigte sich, wie in der Literatur beschrieben, einige Wochen nach erfolgter Intervention [1–3] bei einem Patienten mit den klassischen Risikofaktoren für Vorhofflimmern. Differenzialdiagnostisch für die neurologische Symptomatik sind neben kardioembolischen auch vaskuläre Ursachen in Erwägung zu ziehen. Die ausführliche Anamnese, das neurologische Erscheinungsbild und die Diagnostik mittels Kontrastmittel-CT des Thorax legten schließlich den hochgradigen Verdacht auf diese seltene Krankheitsentität nahe. Obwohl der genaue Mechanismus der atrioösophagealen Fistel umstritten ist, ist der wahrscheinlichste Verletzungsweg eine direkte thermische Schädigung aufgrund der unmittelbaren Nähe des linken Atriums zur Speiseröhre [4–6]. Die Beziehung zwischen dem Ösophagus und der hinteren Wand des linken Vorhofs ist variabel. Die Speiseröhre ist dort am anfälligsten für Verletzungen, wo sie den Bereichen der endokardialen Ablation am nächsten ist [5]. Fisteln wirken funktionell in eine Richtung, von der Speiseröhre zum Vorhof, was für die beobachteten Symptome und Bildgebungsbefunde verantwortlich ist. Klinische Relevanz hat v. a. die unkontrollierte Extravasation von Luft, Speichel, Nahrungsbestandteilen und Bakterien in das Mediastinum, welche Infektionen, Embolien und andere Komplikationen verursachen kann.

Als weitere Ursachen der Fistelbildung werden eine Aggravation des Säurerefluxes aufgrund beschädigter Äste des N. vagus und die daraus resultierende Ischämie durch die Kauterisierung der Endarteriolen diskutiert [5]. Interessanterweise bleibt der linke Vorhof relativ verschont von der direkten Nekrose oder später Perforation durch die Ablation, und es sind keine Berichte über die Verletzung des Vorhofs allein bekannt. Vielmehr kann eine Verletzung der Speiseröhre mit Geschwürbildung zu Erosion und Fistel führen, die schließlich in einigen Fällen in das linke Atrium eindringt [5–7]. Die Ösophagusläsion kann in Abhängigkeit von der Anatomie des Patienten zuerst oder allein mit dem Perikard kommunizieren und eine isolierte atrioperikardiale Fistel bilden. Wenn die Kommunikation durch die Vorhofwand fortgesetzt wird, kommt es zu einer atrioösophagealen Fistel [5].

Aufgrund der selbst bei angemessener Erkennung und Therapie der atrioösophagealen Fistel mit einer beschriebenen Mortalität von über 30 % in den meisten Studien heben Bodziock et al. hervor, dass der Prävention durch bewusste Sedierung, Low-Power-Ablation, Low-Flow-Spülung, engmaschige Ösophagustemperaturmessung und pharmakologische Prophylaxe mit Protonenpumpeninhibitoren oder Histamin-2-Rezeptor-Blockern oberste Priorität zukommt [4]. Über den letalen Verlauf nach Hochfrequenzablation des linken Vorhofs und der Lungenvenen zur Behandlung von Vorhofflimmern bei einem 43-jährigen Mann, der eine atrioösophageale Fistel ausbildete, berichteten Rajapaksha et al. [8].

Wegen der Seltenheit der atrioösophagealen Fistel bleiben die Bewertung und Validierung von Strategien zur Reduzierung eine Herausforderung. Eine zielgerichtete und adäquate Therapie sollte immer im Einklang der verschiedenen Fachabteilungen erfolgen; individualisiert können unterschiedliche Ansatzpunkte gewählt werden. In einem rezenten Review evaluierten Povey et al. [1] Symptome, diagnostische Modalitäten und das Management der atrioösophagealen Fistel. Das Durchschnittsalter der Patienten betrug 61 Jahre, sie waren vornehmlich männlich (73 %), und die Begleitsymptome waren auch hier Fieber in 75 % und neurologische Dysfunktionen in 77 % der Fälle. Als mediane Zeit von Ablation bis zum Eintritt der Symptome wurden 21 Tage (Quartilsabstand: 12–28) beschrieben. Die empfindlichste Thoraxbildgebungsmodalität war die CT (*n* = 135/153; 90 %). Dies fanden auch Han et al. [7], in der in 68 % der Fälle die CT des Brustkorbes zwar die häufigste Diagnosemethode war, sie in 7 Fällen bei primär unauffälligem Befund jedoch wiederholt werden musste. Zu den eindeutigen Bildgebungsergebnissen gehörten freie Luft im Mediastinum (Inzidenzrate 81,73 %) und die Luftembolie des Gehirns (Inzidenzrate 57,53 %) [9]. Diese „klassischen“ bildmorphologischen Besonderheiten zeigten sich auch bei unserem Patienten. Dies sollte in dieser Kombination und der Vorgeschichte nach Pulmonalvenenablation zwingend an die Diagnose einer atrioösophagealen Fistel denken lassen. Bei 19 % der Patienten trat eine sofortige Verschlechterung bei Ösophagogastroduodenoskopien (ÖGD) auf [1]. Diese Manipulationen sollten in jedem Fall vermieden werden, da dadurch induzierte Luftembolien zu erheblichen neurologischen Schäden führen können. Die Sterblichkeit in der von Povey et al. betrachteten Patientenklientel war niedriger bei Patienten, die operiert wurden (39 %), im Vergleich zu endoskopischen Eingriffen (94 %) oder konservativer Behandlung (97 %). Patienten, bei denen eine Vorhofreparatur in Kombination mit einer Ösophagusreparatur oder Ösophagektomie durchgeführt wurde, überlebten mit größerer Wahrscheinlichkeit als diejenigen, bei denen eine Vorhofreparatur allein durchgeführt wurde (OR 6,97; *p* < 0,001). Die Isolierung des ösophagealen Aspekts der Fistel verlieh einen zusätzlichen Überlebensvorteil (OR 5,85; *p* = 0,02) [1].

Jehaludi et al. [2] führten 2018 eine umfassende Literaturrecherche von 65 veröffentlichten Fällen atrioösophagealer Fisteln nach Katheterablation bei Vorhofflimmern durch, mit dem Ziel, die mit den therapeutischen Modalitäten verbundenen Mortalitätsraten zu identifizieren und die adäquateste und risikoärmste Behandlung zur Verringerung der Sterblichkeit vorzuschlagen. Die Autoren kamen zu dem Ergebnis, dass Patienten, die sich einer chirurgischen Reparatur unterzogen, eine 12-mal höhere Überlebenschance als Patienten hatten, die mit Stent-Implantation, Antibiotikatherapie oder keiner Intervention behandelt wurden (*p* < 0,001). Basierend auf dieser Beobachtung glauben die Autoren, dass eine sofortige chirurgische Korrektur der Fistel als Standardbehandlung in Betracht gezogen werden sollte. In der von Jehaludi betrachteten Kohorte [2] zeigte sich eine typische Symptomtrias aus Fieber (52 %), neurologischen Symptomen (38 %) und Hämatemesis (21 %), gefolgt von Brustschmerz (19 %), verändertem Bewusstsein (18 %) und Krampfanfällen (12 %).

Eine vergleichende Untersuchung von chirurgischer Fistelreparatur (*n* = 4) und Oesophagus-Stenting (*n* = 5) führten Mohanty et al. durch [3], und auch sie kamen zu dem Konsens, dass die sofortige chirurgische Reparatur für das Überleben von Patienten mit atrioösophagealer Fistel von entscheidender Bedeutung ist. Alle 5 Patienten, die einen Ösophagus-Stent erhielten, starben innerhalb einer Woche nach dem Eingriff an einer Hirnembolie, einem septischen Schock oder einem Atemversagen. Andererseits waren die 4 Patienten, die eine chirurgische Reparatur erhielten, nach einer mittleren Nachbeobachtungszeit von 2,1 Jahren am Leben (*p* = 0,005).

Aus den USA berichtete Garg 2016 [10] von den häufigsten gastrointestinalen Komplikationen im Zusammenhang mit Vorhofflimmerablationseingriffen, welche im Wesentlichen Gastroparese, thermische Läsionen und Ulzera der Speiseröhre und atrioösophageale Fisteln umfasste. In ihrer Analyse geben die Autoren einen Überblick über das klinische Erscheinungsbild, die Ätiologie, Pathogenese, Prävention und Behandlung dieser Erkrankungen.

In dem beschriebenen, speziellen Fall entschieden wir uns im Konsens, auch in Anbetracht des jungen Lebensalters des Patienten, für ein primär operatives Vorgehen. Die mit einer atrioösophagealen Fistel nach Pulmonalvenenisolation verbundenen Sterblichkeit ist hoch, auch nach korrigierenden Eingriffen [5]. Einen Goldstandard für das therapeutische Vorgehen gibt es nicht. Die Therapie sollte sorgfältig interdisziplinär evaluiert und unter Berücksichtigung der Komorbiditäten des Patienten individualisiert gewählt werden.

## Fazit

Fieber und neurologische Symptome im Zusammenhang mit einer kürzlich erfolgten Ablation sollten zwingend an eine atrioösophageale Fistel denken lassen. Aufgrund des Risikos einer katastrophalen Luftembolie oder massiven Blutung muss eine Instrumentierung des Ösophagus vermieden werden. Das interdisziplinäre Management sollte individualisiert unter Betrachtung des Lebensalters und der Komorbiditäten erfolgen.

## References

[1] Povey HG, Page A, Large S (2022). Acquired atrioesophageal fistula: Need it be lethal? Sizing up the problem, diagnostic modalities, and best management. J Card Surg.

[2] Jehaludi A (2018). Retrospective review of 65 atrioesophageal fistulas post atrial fibrillation ablation. Indian Pacing Electrophysiol J.

[3] Mohanty S (2014). Outcomes of atrioesophageal fistula following catheter ablation of atrial fibrillation treated with surgical repair versus esophageal stenting. J Cardiovasc Electrophysiol.

[4] Bodziock GM, Norton CA, Montgomery JA (2019). Prevention and treatment of atrioesophageal fistula related to catheter ablation for atrial fibrillation. J Innov Card Rhythm Manag.

[5] Kapur S (2017). Esophageal injury and atrioesophageal fistula caused by ablation for atrial fibrillation. Circulation.

[6] Nair GM (2014). Atrioesophageal fistula in the era of atrial fibrillation ablation: a review. Can J Cardiol.

[7] Han HC (2017). Atrioesophageal fistula: clinical presentation, procedural characteristics, diagnostic investigations, and treatment outcomes. Circ Arrhythm Electrophysiol.

[8] Rajapaksha WR, Cunningham KS, Rose TH (2014). A fatal case of atrioesophageal fistula following radiofrequency ablation of left atrium and pulmonary veins for atrial fibrillation. Cardiovasc Pathol.

[9] Liu A (2022). Clinical manifestations, outcomes, and mortality risk factors of atrial-esophageal fistula: a systematic review. Cardiology.

[10] Garg L (2016). Gastrointestinal complications associated with catheter ablation for atrial fibrillation. Int J Cardiol.

